# Evaluation and Correlation of Bite Force With Electromyography (EMG) Activity Before and After Rehabilitation of the First Molar With an Implant-Supported Prosthesis

**DOI:** 10.7759/cureus.31085

**Published:** 2022-11-04

**Authors:** Sheetal R Khubchandani, Anjali Bhoyar, Seema Sathe

**Affiliations:** 1 Prosthodontics, Sharad Pawar Dental College and Hospital, Datta Meghe Institute of Medical Sciences, Wardha, IND

**Keywords:** first molar, masticatory efficiency, implant therapy, emg, maximum bite force

## Abstract

Background

Prevalence of tooth loss significantly increases as age advances. The permanent first molar is regarded as the key to proper occlusion and, thus, its loss causes significant deterioration of the functioning of the masticatory apparatus and overall stomatognathic system. It not only affects the occlusal integrity but also the activity of surrounding tissues and muscles.

Aim and objectives

To evaluate and correlate the maximum bite force (MBF) and electromyography (EMG) activity before and after 24 hours and two months of rehabilitation of the missing permanent first molar with single-implant crowns.

Materials and methodology

This observational, prospective in-vivo type of study was done on 10 patients receiving dental implants for missing molars.

Results

There was a significant correlation between bite force and EMG activity post-treatment.

Conclusion

Dental implant therapy can be very well utilized for the replacement of single missing teeth.

## Introduction

There are several treatment modalities available to rehabilitate missing molars, such as removable dental prostheses (RDPs), fixed dental prostheses (FDPs), or dental implants. Removable dental prostheses for single-tooth replacement may prove uncomfortable and not preferable in terms of wear and function [1]. FDP by preparation of adjacent natural teeth can also be advocated. The advent of implants and their high success rates and the fact that they do not need tooth preparation of vital tooth adjacent to the missing teeth becomes the reason for choosing it as a preferable treatment option [2]. The bite force is a marker of the health of the system of mastication and its functional state. Maximum bite force (MBF) levels are due to a combination of the action of the muscles of elevation of the jaw. Also, it can be modified by factors such as biomechanics of the jaw and reflex mechanisms that are present in the body. It is used for evaluating the performance of various prostheses [3]. Various devices or techniques can be used to measure bite force. Strain gauges, transducers, or pressure-sensitive sheets are commonly used in routine dental practice. Other advanced modalities such as electromyography (EMG), ultrasound sonography (USG), or T-scans are also used to assess various parameters of masticatory efficiency. Electromyography or EMG activity is one of the modalities that is used commonly. This advanced technique measures and evaluates the muscle activity of the skeletal muscles. It can be used to diagnose and treat formulations of conditions such as para-functional habits like bruxism and syndromes like myofascial pain dysfunction syndrome (MPDS). Its applications in prosthetic dentistry have remained somewhat unexplored. EMG can be used to assess and compare the functioning of various prostheses [4]. The bite force is maximum in the first molar area (300-600 N) and so the loss of first molar support can result in the reduction of the resultant bite force [5]. Ranjan in 2020 has conducted a study to evaluate MBF in the molar and incisor areas. Subjects with normal occlusion and systemically healthy were taken into consideration. They also determined the impact of age and gender on MBF. The results showed that the mean MBF was 122 N in the incisor area and 320N in the first molar area. In a few subjects, higher values of bite force were incident. The author concluded that measurement of the bite force of an individual should be done routinely. It is helpful in assessing the prognosis and success of prostheses. The author also stated that future research is needed to know the influence of prosthesis type in the assessment of MBF [6]. Various options for prosthetic rehabilitation are available to rehabilitate chewing function in partially edentulous patients.

Studies comparing prostheses to determine which is best suitable use biting force as one of the parameters. Few studies [7,8] have studied the influence of prosthodontic rehabilitation on mastication in subjects with few missing teeth. The findings of those studies were controversial. Kapur concluded that removable dental prostheses (RDPs) and partial implant fixed dental prostheses (PI-FDPs) gained the same amount of chewing efficiency. On the contrary, Leidberg showed higher values of the chewing index in patients with a fixed dental prosthesis than in removable dental prosthesis wearers. Impairment in masticatory function can have a negative impact on the quality of life (QoL) of the patient. Hence, the effects of different prostheses on mastication are important to determine [9]. In literature, implant therapy is increasingly utilized to restore partial edentulism and there is evidence of improvements in mastication using implant-supported prostheses when compared to tooth-supported [10]. Teeth and jaw muscles both function in synergy to produce a balance in occlusion. However, it is disturbed when teeth or the jaw muscles or muscles of mastication deviate from the usual physiology. Measurement of masseter muscle thickness has been regarded as a marker of the functioning of muscle [11]. MBF measurement was accomplished by utilizing a customized fabricated bite force gauge. It was manufactured using various engineering devices such as strain gauges, load cells, microcontrollers, amplifiers, plate connectors, and computer software. The mechanism of action used was analog-to-digital conversion. The pressure or force sensitivity is highly precise and accurate. Software was used to display the readings of the patient while clenching in maximum intercuspation. The measured bite force was in newton and, along with it, the extent of squeeze or touch, i.e., light, medium, and heavy, can also be plotted on a graph or seen on the monitor.

## Materials and methods

This observational, prospective in-vivo type of study was initiated after the ethical approval obtained from the Datta Meghe Institute of Medical Sciences (DU) Institutional Ethics Committee at the Department of Prosthodontics and Crown and Bridge, Sharad Pawar Dental College and Hospital, Datta Meghe Institute of Medical Sciences (Deemed to be University), Sawangi (Meghe), Wardha. IRB approval no is DMIMS(DU)/IEC/Aug-2019/8271.

The sample size calculated was 10 subjects in each group. Patients were explained about the procedure and those willing for implant therapy were included and the informed written consent form was signed. Patients with missing maxillary or mandibular/left or right permanent first molars irrespective of age and gender were included. The opposite arch should be intact with adequate bone width and height for implant placement and healthy periodontium surrounding adjacent natural dentition. Patients with a habit history of smoking or other para-functional habits, acute conditions such as infections, and uncontrolled systemic conditions were excluded.

**Figure 1:**
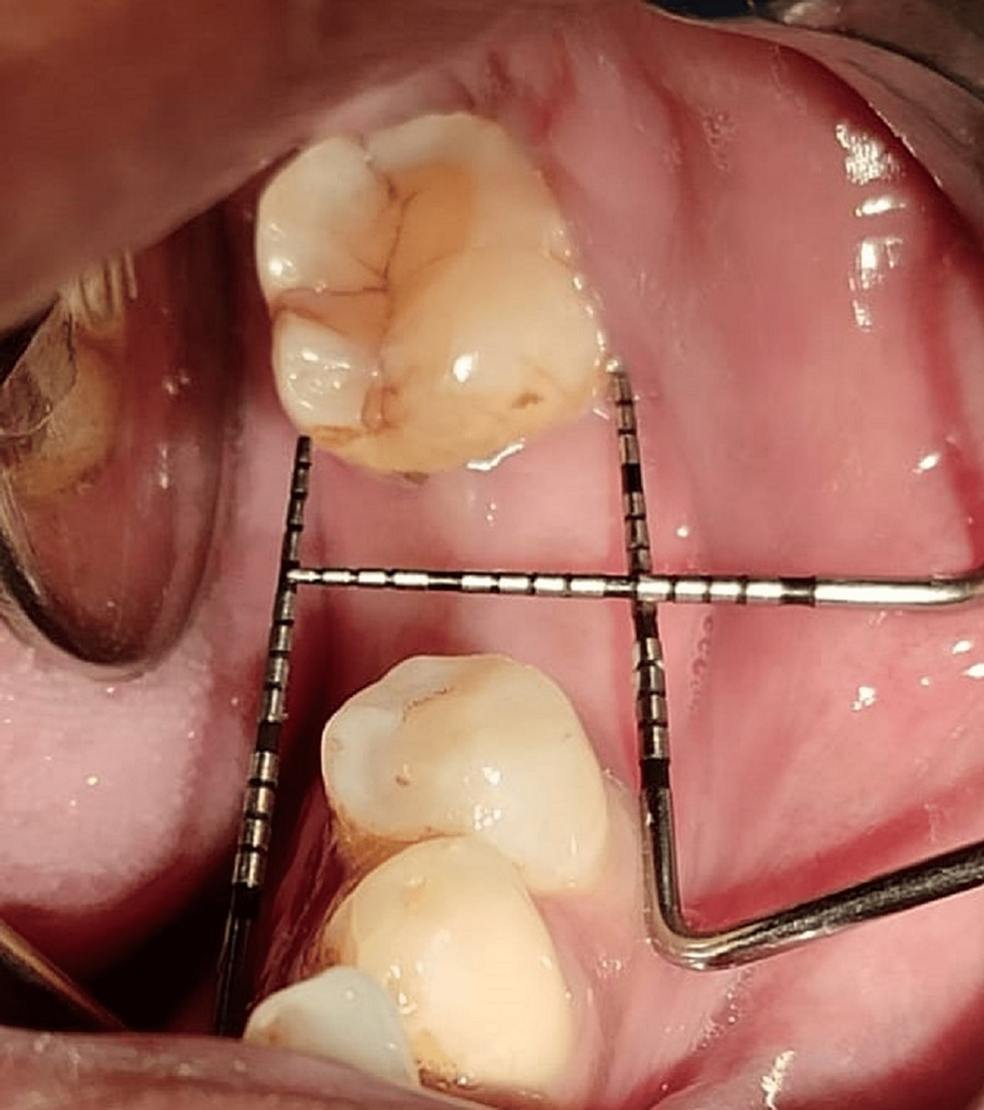
Clinical examination.

Following procedures were carried out. A complete clinical examination was done (Figure 1).

**Figure 2:**
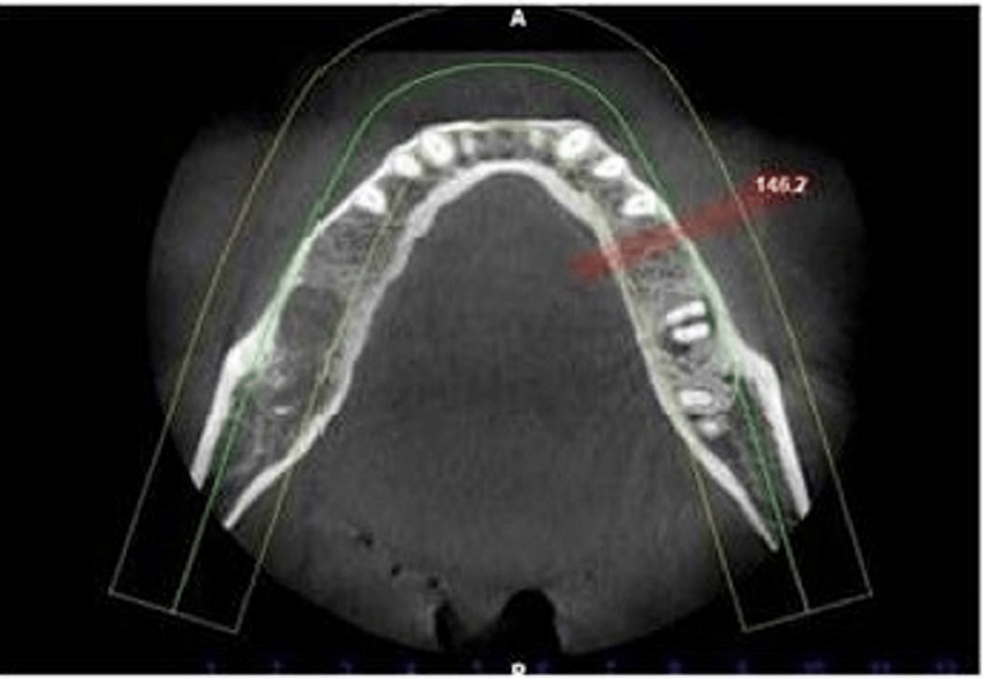
Radiological examination.

Radiological examination was done to assess the availability of space and for evaluation of bone height (apico-coronal) and width (bucco-lingual) availability (Figure 2). 

Diagnostic impressions were made. An interim partial denture was fabricated and given with a missing molar for ease of placement of the bite force gauge and recording. MBF and EMG were recorded preoperatively.

Recording of MBF

**Figure 3:**
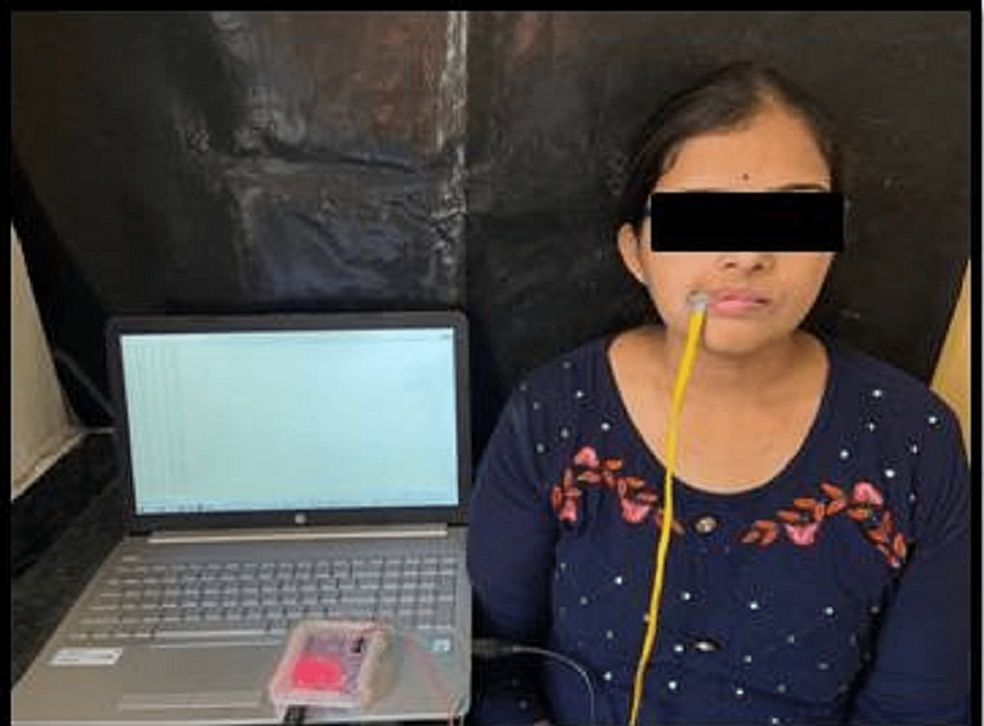
Recording of MBF. MBF: maximum bite force.

MBF was recorded utilizing a customized bite force gauge. The sensor chip was first covered with a plastic sleeve to avoid contamination from one patient to another. Readings of the bite force visualized on the computer screen in the form of numerical as well as graphical data were recorded. MBF was then noted for the patient (Figure 3). 

Recording of EMG

**Figure 4:**
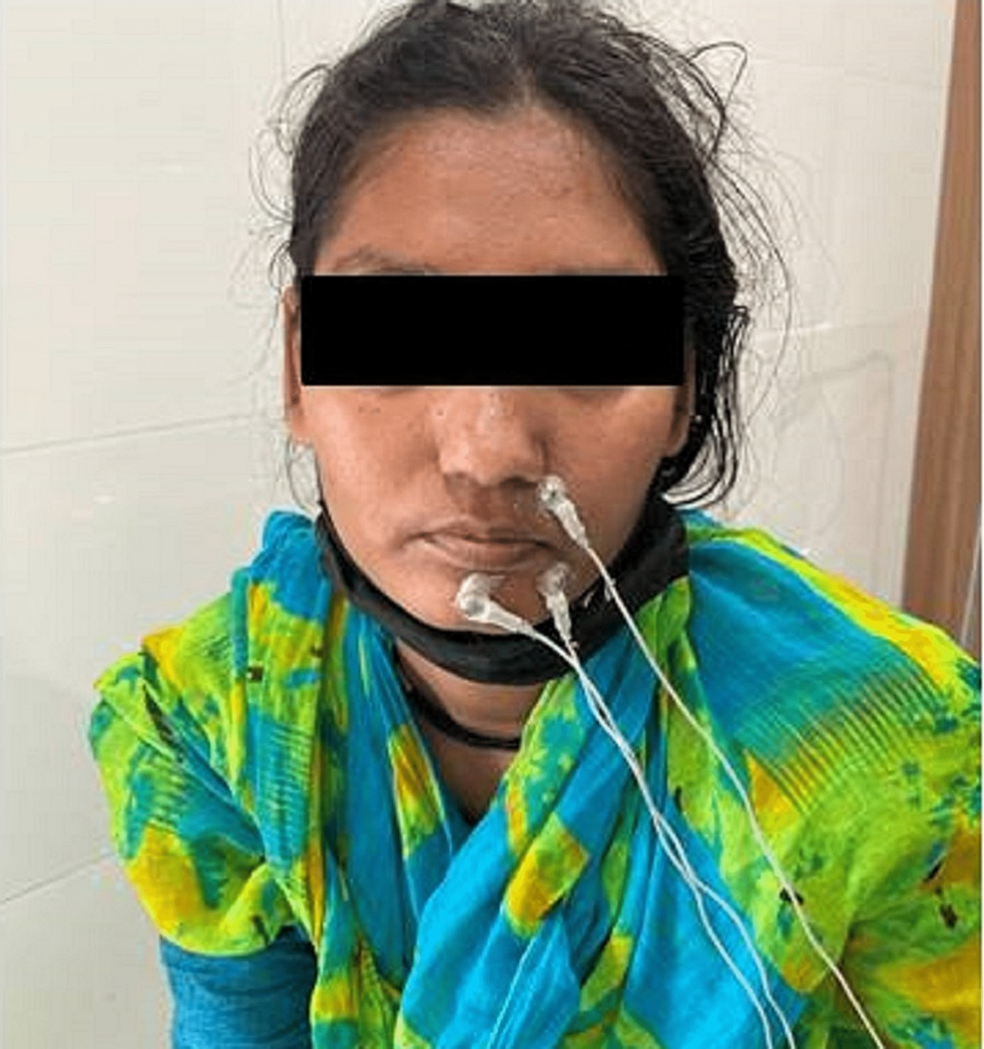
Recording of EMG activity. EMG: electromyography.

Electrodes were attached to the face in the region of the masseter muscle. Current and amplitude values were set according to the needs of the physiologist. EMG activity of the masseter muscle on an EMG machine was recorded on a computer screen (Figure 4).

**Figure 5:**
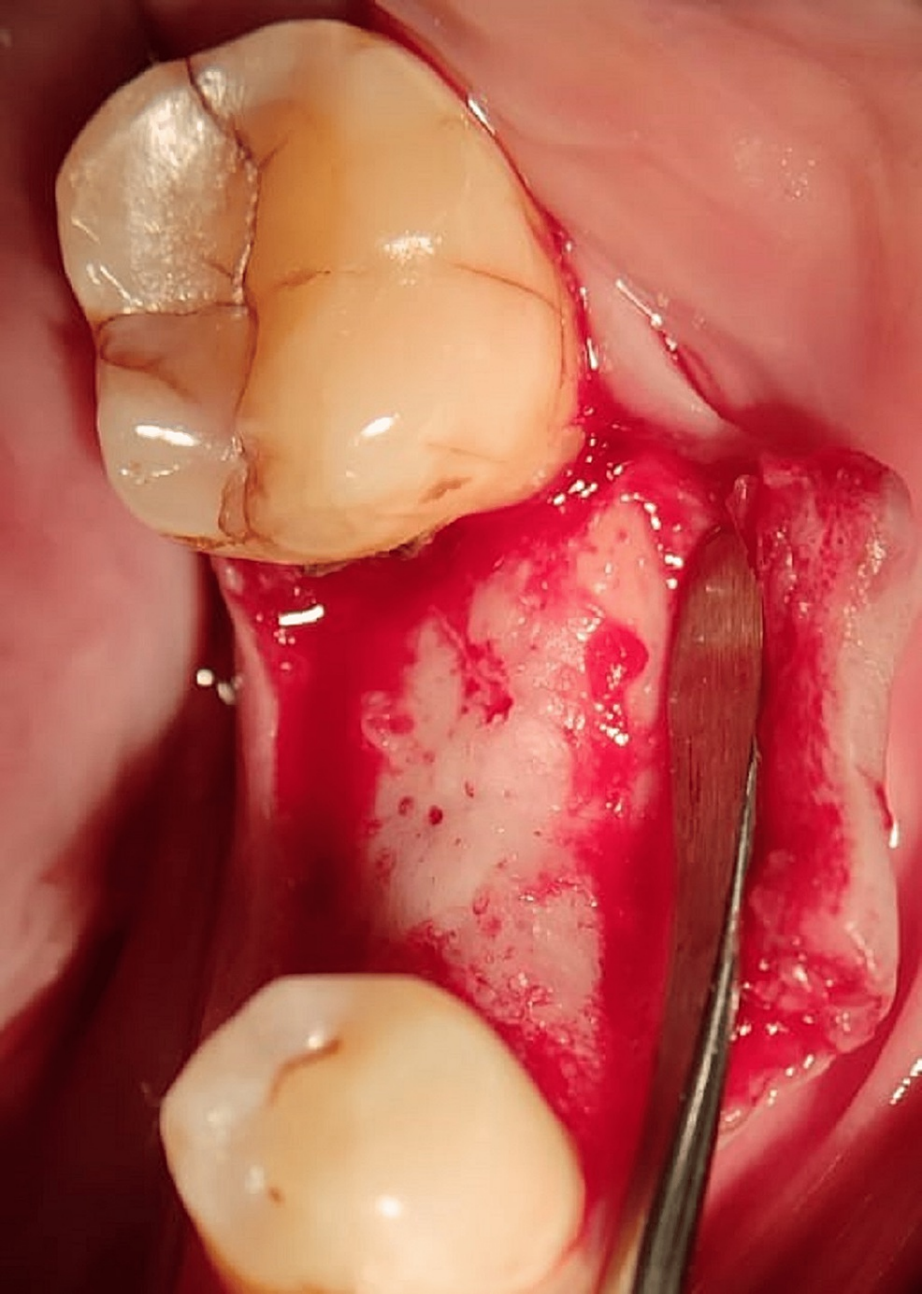
Incision and reflection of the flap.

Implant placement was done using a two-stage surgical technique. Part preparation and sensitivity test for local anesthesia were done. Local anesthesia was injected. The crevicular incision was given and the flap was reflected, and drilling for the implant placement was done sequentially according to the size of the implant used using the Osstem Implant Taper Kit (Osstem & Hiossen Inc., Huntingdon, UK) (Figure 5).

**Figure 6:**
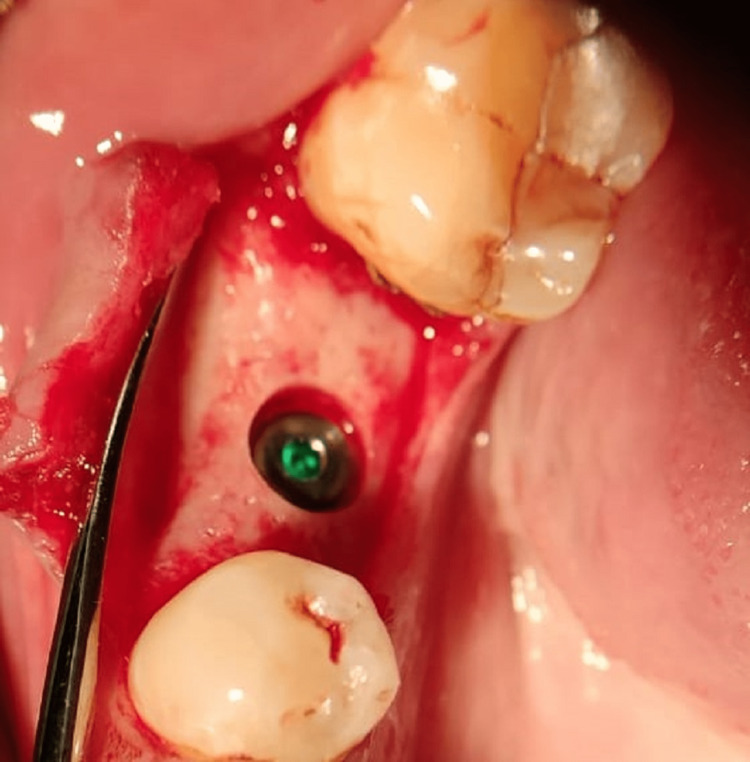
Implant placement.

Implant placement was completed and the cover screw was attached, followed by suturing (Figure 6).

**Figure 7:**
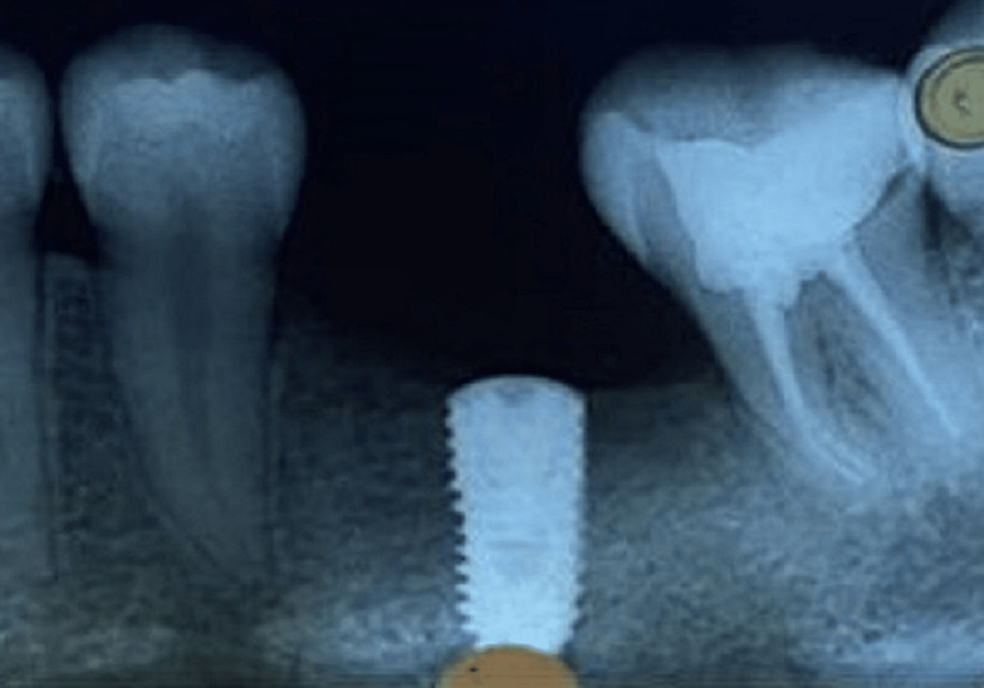
Radiological examination for osseointegration.

After three months, the radiographic evaluation of the placed implants for proper osseointegration is done (Figure 7).

Local anesthesia followed by an incision was given and the flap was reflected to expose the cover screw. The cover screw was removed and the gingival former was placed. After the placement of the gingival former, two weeks of healing period was provided.

The patient was evaluated for complete healing and formation of the gingival collar after placement of the gingival former. The gingival former was then removed, and transfer/pick-up impression copings were attached intra-orally on the implant fixture. Implant-level closed tray impressions were made using the putty wash impression technique. A lab analog was attached to the impression, and a gingival mask was adapted. A cast was poured. A VITA classic shade guide was used to assess the shade of the patient’s teeth. The final crown prosthesis was then cemented.

MBF was measured after 24 hours of cementation of the single-implant-supported crown prosthesis. Also, EMG activity was assessed. MBF was measured again after two months of follow-up of the cementation. Also, EMG activity was assessed. Mean MBF and EMG were calculated. Data entry was done in Microsoft Office Excel (Microsoft Corp., Redmond, WA) and then analysis of the data was done.

## Results

Statistical analysis was done using analytical and descriptive statistics. The tests used were Shapiro-Wilk test and paired t-test. Analysis was done using the software Statistical Package for Social Sciences (SPSS) Version 24.0 (IBM Corp, New York, USA).

A total of 10 subjects that fulfilled the inclusion criteria were included in the study. Results showed that the mean age of the study population was 47.00 ± 9.40 with a range of 36 to 62 years. There were 3 (30.0%) males and 7 (70.0%) females. It was seen that the prevalence of missing permanent first molars was more in females (Table 1).

**Table 1:** Demographic details (age). n=sample size/number of participants, SD: standard deviation.

Age (n=10)	Mean	SD	Range
36-62 Years	47.00	9.40	36-62

The mean MBF before and after 24 hours of cementation with a single-implant crown was compared. The mean MBF before cementation (324.30 ± 79.71) was significantly increased (p<0.001) after 24 hours of cementation (407.30 ± 63.65) (Table 2). 

**Table 2:** Comparison of mean MBF before and after 24 hours MBF: maximum bite force, SD: standard deviation, SE: standard error, MD: mean deviation, CI: confidence interval, t-value: test value, p-value: probability value. #P-value derived from paired t-test; †significant at p<0.05.

MBF	N	Mean	SD	SE	MD	95% CI	t-value	P-value^#^
Before	10	324.30	79.71	25.20	−83.00	−106.25−59.74	−8.074	<0.001^†^
24 Hours	10	407.30	63.65	20.12				

The mean MBF before and after two months of cementation with a single-implant crown was compared. The mean MBF before cementation (324.30 ± 79.71) was significantly increased (p<0.001) after two months of cementation (457.70 ± 49.02) (Table 3). 

**Table 3:** Comparison of mean MBF before and after two months. MBF: maximum bite force, SD: standard deviation, SE: standard error, MD: mean deviation, CI: confidence interval, t-value: test value, p-value: probability value. #P-value derived from paired t-test; †significant at p<0.05.

MBF	N	Mean	SD	SE	MD	95% CI	t-value	P-value^#^
Before	10	324.30	79.71	25.20	−133.40	−171.36−95.43	−7.948	<0.001^†^
Two months	10	457.70	49.02	15.50				

The mean electromyography activity before and after cementation (24 hours) with a single-implant crown was compared. The mean EMG activity before rehabilitation (141.47 ± 30.08) was significantly increased (p<0.001) after 24 hours of rehabilitation (169.81 ± 26.40) (Table 4). 

**Table 4:** Comparison of mean EMG activity before and after 24 hours. EMG: electromyography, SD: standard deviation, SE: standard error, MD: mean deviation, CI: confidence interval, t-value: test value, p-value: probability value. #P-value derived from paired t-test; †significant at p<0.05.

EMG	N	Mean	SD	SE	MD	95% CI	t-value	P-value^#^
Before	10	141.47	30.08	9.51	−28.34	−43.76−12.91	−4.156	0.002^†^
24 Hours	10	169.81	26.40	8.35				

The mean electromyography activity before and after cementation (two months) with a single-implant crown was compared. The mean EMG activity before rehabilitation (141.47 ± 30.08) was significantly increased (p<0.001) after two months of rehabilitation (204.56 ± 41.13) (Table 5).

**Table 5:** Comparison of mean EMG activity before and after two months. EMG: electromyography, SD: standard deviation, SE: standard error, MD: mean deviation, CI: confidence interval, t-value: test value, p-value: probability value. #P-value derived from paired t-test; †significant at p<0.05.

EMG	N	Mean	SD	SE	MD	95% CI	t-value	P-value^#^
Before	10	141.47	30.08	9.51	−63.09	−76.94−49.23	−10.302	<0.001^†^
24 Hours	10	204.56	41.13	13.00				

Pearson’s correlation test did not show any significant (p=0.164) correlation between MBF and EMG after 24 hours of cementation. Pearson’s correlation test did not show any significant (p=0.611) correlation between MBF and EMG after two months of cementation (Table 6).

**Table 6:** Correlation of MBF and EMG. EMG: electromyography, MBF: maximum bite force, R-value: correlation value, P-value: probability value. #P-value derived from paired t-test.

Correlation	R-value	P-value^#^
10	0.477	0.164
10	0.184	0.611

## Discussion

Dental implants are increasingly used nowadays to replace a single or multiple or full set of missing permanent dentitions of the patient. Increasing popularity is owing to its advantages over conventional RDPs and FDPs are improved function and conservation of the natural tooth structure of the adjacent teeth [1,2]. Various authors studied the survival rate [12,13] and prognosis of implant-supported restorations over fixed dental prostheses. They suggested significant differences, and the prognosis was excellent with implant-supported restorations [14]. According to a systematic review [15], the implant success and survival rates of single-implant crown prostheses were approximately 94.3% and 95.6%, irrespective of the method of retention and type of support used for the prosthesis, after examination at a long interval of 72 months. The prosthesis success rate was 85.4%. Similar results that the cumulative success rate was 95% after 24 months in a study by Becker et al. [16].

The analysis of the samples depicted that the mean age of the partially edentulous patients was 47 with a range of 36 to 62, which is a widely distributed age range. So, implant placement can be advocated irrespective of age and gender, provided that all the other systemic factors are in control. So, similar inclusion criteria were followed in the present study. Also, we have included missing first molars of any jaw, and the implant placement position was not specified. Maxillary or mandibular, right or left, all the implants were included. Similarly, Naert et al. stated that the treatment success of implants is independent of the jaw size, position, etc. [17].

In the current study, opposite arches were chosen that were intact. This ensured the recording of the bite force by proper placement of the sensor. Also, the missing molar area, which is an edentulous space was temporarily restored using an acrylic partial denture so as to easily record the bite force. The bite force recording gauge was indigenously fabricated for the study.

The capacity of dental implants to resist the functional force of chewing may exceed that of the teeth. This functional force can be called "masticatory force" or "bite force," which is the force applied on the masticatory apparatus during the function of chewing or others. MBF implies its maximum value when performing functions such as clenching of the teeth in the maximum intercuspation position. MBF has been studied by many authors and is considered a potent indicator for the functioning of the masticatory system/apparatus [3]. Some authors studied it bilaterally [18] and some took only unilateral readings [19]. Both ways, results were variable and, at the same time, satisfactory and statistically significant when compared to the baseline. Only a few studies were there that compared both the ways that are unilateral and bilateral at the same time [20].

van der Bilt et al. [20] compared MBF and muscle activity in 81 healthy individuals. The author compared unilateral as well as bilateral bite forces and muscular activity during maximum clenching function. The results from this study can be used as a standard normal value to compare them with readings from other patients during other studies. The author observed that in 81 healthy individuals, bilateral MBF was 569 N, while unilateral recordings were done and the results showed that MBF was lower than that of bilateral recordings. It was 430 N on the one side while 429 N on the other side. The author also concluded that there was no significant statistical difference in masseter muscle activity in unilateral clenching when compared to bilateral one. Temporalis muscle activity showed little change.

Observations of the current study revealed that the mean MBF prior to treatment of a missing first molar was 324 N (which is in the normal range of 300-600 N) [5], and it significantly increased post-treatment almost by 83 N. The mean MBF post-24-hour treatment and post-two-month treatment was 407 N and 457 N, respectively. Also, the change in mean MBF after two months was 133 N when compared to the baseline and 50 N when compared to the 24-hour readings. Similar findings that there is an increase in bite force after implant crowns were seen in a study by Zou et al. [21], which showed an increase of only 3-6% but was statistically significant when statistics were done. It was 3.31% for a unilateral single posterior implant and 6.83% for a bilateral restoration. Also, in a study by Ferrario et al. [19], they concluded that bite force in men is higher than in women; similar results were observed in the current study.

The mean unilateral EMG activity of the masseter muscle in the region of the missing first molar was 141 microvolts before any treatment was done for the missing tooth. The mean unilateral EMG was 169 microvolts after treatment done using a single-implant crown prosthesis. A significant increase in EMG activity was seen at almost 28 microvolts in 24 hours post-cementation of the single-implant crown prosthesis. Also, after two months, it was found that the mean EMG activity was 204 microvolts which significantly increased by almost 63 microvolts. So it can be concluded that there is improvement in muscle function and thus in masticatory performance after rehabilitation of one occluding pair of molars. Gartner et al. [22] have revealed similar results in their study. The EMG recordings in the study revealed that an unusual pattern is observed in patients in whom implants are placed.

The mechanism that leads to improved MBF and muscle EMG activity is that an increase in the number of occluding pairs improves the masticatory force that is applied, and further, this force acts on the muscles and muscle activity is enhanced. All these factors lead to improved masticatory function. But on the contrary, some studies showed that increases in MBF were not related to muscle function. Both are independent. Hence, no correlation can be manifested from the results. Pearson’s coefficient of correlation did not show any correlation. Neither after 24 hours nor two months of rehabilitation. Similar results were observed in a study by Sathasivasubramanian et al. [11]. The author stated that there wasn’t any significant difference in masseter muscle thickness and thus activity. It was also added that the masseter muscle undergoes atrophy when teeth are lost. So, independent improvements can be seen in masticatory function and muscle activity, but both cannot be well correlated. Also, Saifuddin et al. [23] concluded muscle action and biting force are independent when records are to be taken. The biting force reference does not influence muscle action but gives a false estimation.

Proeschel et al. [24] in a study revealed that the mean chewing force recorded was 220 N, while when the estimation of the EMG was done, it was 273 to 475 microvolts in nine patients. Recordings were made unilaterally as that of the present study. So, the author quoted that their study indicated different activity and force characteristics.

Limitations and scope of the study; in the current research, two parameters, namely, biting force and EMG activity of the muscle, were taken into account, while the masticatory efficiency is multi-factorial [25]. So, the scope to include other parameters as well remains. A follow-up period of more than two months or a yearly follow-up can be done. A longitudinal study can be performed on the same study population. Large sample size and retrospective data can also be added to increase the significance of the results.

## Conclusions

The mean MBF and EMG activity were in their usual range before the cementation of single-implant crowns. The mean MBF and EMG activity were significantly increased immediately (24 hours) and at an interval of two months after the cementation of single-implant crowns.

The study provides insight for patients as well as clinicians to choose the best possible treatment option for partially edentulous situations. The results of the study can be utilized for patient education and awareness regarding the improvement in mastication after implant therapy. The customized bite force gauge that is used in the study can opt for an easy and convenient alternative option to measure the bite force in a more economic way.
